# Combining label-free Raman spectroscopy with machine learning to monitor COVID-19 disease from acute infection to recovery

**DOI:** 10.1117/1.JBO.31.7.077002

**Published:** 2026-07-20

**Authors:** Maryam Heidarifard, Frédéric Leblond, Frédérick Dallaire, Elsa Brunet-Ratnasingham, Nassim Ksantini, Myriam Mahfoud, Guillaume Sheehy, Hugo Soudeyns, Philippe Jouvet, Sze Man Tse, Caroline Quach, Daniel E. Kaufmann, Katherine Ember, Mathieu Dehaes

**Affiliations:** a Centre de recherche Azrieli du CHU Sainte-Justine, Montreal, Quebec, Canada; b CHU Montreal, Research Centre, Montreal, Quebec, Canada; c Université de Montréal, Institute of Biomedical Engineering, Montreal, Quebec, Canada; d Polytechnique Montreal, Department of Engineering Physics, Montreal, Quebec, Canada; e Université de Montréal, Department of Microbiology, Infectiology and Immunology and Department of Pediatrics, Faculty of Medicine, Montreal, Quebec, Canada; f Emory University School of Medicine, Pathology Advanced Translational Research Unit (PATRU), Atlanta, Georgia, United States; g Université de Montréal, Department of Pediatrics, Montreal, Quebec, Canada; h Lausanne University Hospital and University of Lausanne, Division of Infectious Diseases, Vaud, Switzerland; i Université de Montréal, Department of Medicine, Montreal, Quebec, Canada; j Université de Montréal, Department of Radiology, Radio-oncology and Nuclear Medicine, Montreal, Quebec, Canada

**Keywords:** COVID-19, label-free Raman spectroscopy, machine learning modeling, plasma, biomolecular signature, disease progression

## Abstract

**Significance:**

Monitoring COVID-19 disease from acute infection to recovery is critical to understand biochemical dysregulation and COVID-19 heterogeneity over time.

**Aim:**

Our aim is to develop an approach combining label-free Raman spectroscopy and machine learning modeling to enable sensitive biomolecular detection of COVID-19 over time.

**Approach:**

Hospitalized patients infected with severe acute respiratory syndrome coronavirus 2 (SARS-CoV-2) were recruited and stratified based on respiratory support (critical and non-critical). Controls had a negative SARS-CoV-2 test. Blood was collected in the acute and recovery phases and was analyzed with Raman spectroscopy. Four machine learning models based on Raman spectra were developed to differentiate critical and non-critical patients in the acute and recovery phases from controls. For each group of patients, two additional models also classified the patient status (acute versus recovery) using cross-sectional and longitudinal analyses.

**Results:**

Raman peaks assigned to proteins, glucose, fatty acids, lactic acid, vitamin A, and lipids were identified in models. Overall, area under the receiver operating characteristic curve values were between 0.83 and 1.00 with sensitivities, specificities, and accuracies between 73% and 100%, 77% and 100%, and 78% and 100%, respectively.

**Conclusions:**

These results highlight the capability of combined Raman spectroscopy and machine learning modeling to stratify patients at admission, monitor recovery after discharge, and support strategies to potentially reduce the risk of long-COVID.

## 1 Introduction

COVID-19 is a respiratory disease caused by severe acute respiratory syndrome coronavirus 2 (SARS-CoV-2).^1^^,^^2^ By April 2026, COVID-19 had caused over 7M deaths worldwide, while the long-COVID syndrome currently affects ∼400  M individuals worldwide.^3^^–^^5^ COVID-19 is a heterogeneous disease, with a clinical spectrum ranging from asymptomatic infection to severe illness and death.^6^^,^^7^ Due to the highly dynamic nature of COVID-19, clinical characteristics alone may not be sufficient for stratifying patients during hospitalization.^8^^,^^9^ Notably, some COVID-19 patients with high SARS-CoV-2 viral loads during the acute phase of infection succumbed, while others survived, indicating that additional factors are involved.^10^ The majority of COVID-19 patients recover, whereas a subset progresses to develop severe illness.^11^ Nearly 30% of COVID-19 patients experience a delayed recovery back to their healthy state due to persistent disruptions in metabolic homeostasis.^12^^,^^13^ Hence, there is an interest in monitoring disease progression from acute infection to recovery. A point-of-care method for rapid identification of biochemical changes associated with COVID-19 recovery could provide insights for novel therapeutic strategies, particularly given the growing number of long-COVID cases.^11^

Currently, polymerase chain reaction and enzyme-linked immunosorbent assay are used to diagnose SARS-CoV-2 infection. However, these tests fail to capture biochemical alterations associated with recovery or mortality. Also, they do not enable effective long-term monitoring after the initial infection.^14^^,^^15^ Gold-standard metabolomic techniques have been used to characterize a wide range of biomolecules that differentiate healthy controls from COVID-19 patients, enabling classification of early- and late-stage disease.^16^^,^^17^ However, these tools are costly, non-portable, and require hours of analysis and trained laboratory scientists.^18^^,^^19^

Raman spectroscopy (RS) is an emerging optical technique used to assess the molecular composition (e.g., proteins, amino acids, and lipids) of a sample.^20^ When incident laser light is emitted onto a sample, the inelastically scattered light provides information about molecular bonding and structure, which is visualized as a Raman spectrum.^21^^–^^23^ RS is a high-speed, low-cost, and non-invasive technique that does not rely on chemical reagents nor require complex sample preparation, allowing efficient data acquisition and analysis within minutes from liquid blood plasma samples. Previous studies have used vibrational spectroscopy to detect COVID-19 infection in plasma, serum, and saliva samples.^24^^–^^33^ Many of these studies rely on surface-enhanced Raman spectroscopy (SERS), which is costly and complex.^34^^–^^37^ The only non-SERS Raman study used confocal Raman microspectroscopy, which is slow, costly, and unsuitable for large-scale testing.^28^ Although saliva samples have proven promising for COVID-19 screening, sample manipulations are difficult, and data in intubated patients are limited.^24^^,^^25^^,^^31^^,^^38^

Machine learning modeling (ML) has been widely applied to analyze large RS datasets, demonstrating a potential for COVID-19 detection in saliva, serum, and plasma, achieving accuracies ranging from 0.78 to 0.98.^24^^,^^25^^,^^31^^,^^33^^,^^39^^,^^40^ To our knowledge, RS-ML has not previously been used to monitor COVID-19 disease in hospitalized patients from acute infection to recovery.

In this study, we developed and tested the capability of a label-free RS-ML tool to detect temporal/longitudinal biomolecular changes from acute infection to recovery in hospitalized COVID-19 patients and differentiate them from healthy controls. We hypothesized that monitoring these biochemical patterns may enhance our understanding of how the disease progresses over time, thereby supporting the development of novel therapeutic strategies.

## 2 Material and Methods

### 2.1 Study Design and Participants

Patients with a positive SARS-CoV-2 infection confirmed by reverse transcription polymerase chain reaction (RT-PCR) and admitted to the Centre Hospitalier de l’Université de Montréal (CHUM, Quebec, Canada) during the first wave of the pandemic were enrolled. Patients were initially recruited through the Biobanque québécoise de la COVID-19 (Study No. MP-02-2020-8929, 19.389).^41^ Written informed consent was obtained from all participants. This study was approved by the institutional ethics review board of the CHUM Research Centre. Blood plasma samples were collected at 11±5 days after symptom onset (DSO) following clinical worsening. At this time (acute phase), patients were evaluated by an infectious disease physician and stratified based on the respiratory support need: non-critical disease status was attributed to hospitalized patients requiring no supplemental oxygen (severity score 0) or patients with a mild-to-moderate disease requiring oxygen supplementation via nasal cannula (severity score 1); critical disease status included hospitalized patients requiring non-invasive ventilation (severity score 2) or invasive ventilation via endotracheal intubation (severity score 3) or extracorporeal membrane oxygenation (severity score 4). A subset of participants was contacted after hospital discharge for a follow-up visit (recovery phase) between DSO60 and DSO130, which included blood collection. During the follow-up visit, an infectious disease physician evaluated the participants and confirmed the absence of SARS-CoV-2 symptoms. RT-PCR was not repeated as not clinically indicated. Healthy controls with a negative SARS-CoV-2 test confirmed by RT-PCR were also recruited and underwent blood collection.

### 2.2 Blood Collection

Blood was collected in acid citrate dextrose tubes and processed at CHUM (centrifuged at 2000 rpm for 10 min at room temperature). The plasma was biobanked and stored in a −80°C freezer until RS data acquisition.

### 2.3 Raman Spectroscopy System and Data Acquisition

Raman spectra were acquired and analyzed at the Centre de recherche Azrieli du CHU Sainte-Justine (Montreal, Quebec, Canada) and approved by its institutional ethics review board. The RS system was based on a 785-nm excitation wavelength laser (IPS, Somerset, New Jersey, United States) and inelastically scattered light captured within the spectral range of 600 to $1700\;{\mathrm{cm}}^{-1}$. Average laser power at the sample was 100 mW, providing a high signal-to-noise ratio (SNR) while avoiding sample deterioration. Light was collected using a spectrometer (Wasatch Photonics, Morrisville, North Carolina, United States) equipped with a 1024-pixel charge-coupled device camera and optimized to enhance SNR with short exposure times. The shorter integration times (300 to 1000 ms per measurement) allowed for equivalent Raman signal intensity among samples. RS measurements were acquired at a single spot (spot size at the tip: 170  μm; collection area: ∼1  mm diameter). Plasma samples (100  μL) were loaded into a 400-μL enhanced chemical resistance aluminum cuvette and positioned at 11 mm from the laser probe (nominal numerical aperture of 0.33, f/1.3 collection optics) to reduce noise and minimize signal contamination from cuvette walls. Before data acquisition, the plasma was transferred from dry to regular ice for 30 min followed by 30 min at room temperature. Vortexing was limited to 60 s. Raman signals from 20 measurements were averaged. Liquid plasma samples were used to maintain the accuracy of Raman spectral data.

### 2.4 Raman Spectral Preprocessing

Spectral preprocessing was performed with the following steps: (1) data averaging, (2) cosmic ray artifact removal, (3) baseline subtraction of the autofluorescence signal using the BubbleFill algorithm,^42^ (4) smoothing using a Savitzky–Golay filter of order 3 with a window size of 11,^43^ and (5) standard normal variate normalization.^44^

### 2.5 Feature Selection and Machine Learning Modeling

An L1-regularization support vector machine (SVM) was used to remove irrelevant (e.g., in-between peaks region) and redundant (e.g., Raman peaks consisting of several spectral bins) features and to reduce the dimensionality from 1100 features to 26. These 26 peaks formed a common Raman library and were fitted with Gaussian functions resulting in a reduced space containing the full width at half maximum of these peaks.^45^^–^^47^ The weight of each peak was provided by the L1-regularization SVM and served to rank each feature. Following dimensionality reduction, a gradient boosting model based on decision trees was used for classification due to its efficiency in handling multiple types of data (spectral and demographic or clinical variables) and its ability to capture non-linear relationships while minimizing overfitting through ensemble averaging and regularization.^48^ Classification models incorporated Raman data, age, and sex as input variables. Age and sex were included as they have previously been identified as risk factors for death or critical illness in COVID-19 patients.^49^^,^^50^ Hyperparameters of the classification models were optimized using a grid search over multiple combinations and included class weight, learning rate, number of estimators, minimum loss reduction, L1 and L2 regularization terms, and maximum tree depth. To account for class imbalance among groups of size $N_{1}$ and $N_{2}$ ($N_{1}>N_{2}$), the class weight hyperparameter was defined such that the class with $N_{1}$ samples was assigned a weight of $N_{1}/N_{2}$, whereas the other class was assigned a weight of 1. During data training, this approach increased the penalty term for misclassifying samples from the class with $N_{1}$ samples. A fivefold cross-validation procedure was applied to evaluate the classification performance. The dataset was randomly divided into five equal subsets. In each iteration, 80% of the data were used for training and the remaining 20% for testing. Model performance was averaged across the five folds to ensure robustness. To prevent classification bias, data from the same patient were used either in the training or the testing set during cross-validation, thereby avoiding data leakage. For each classification, the results were reported for the hyperparameters set yielding the highest area under the receiver operating characteristic curve (AUC), along with the corresponding sensitivity, specificity, and accuracy, as well as the confusion matrix. Features that were extracted in three or more of the folds of the fivefold cross-validation of each model were reported with performance metrics.

Eight RS-ML models were developed and classified into the following groups: critical patients in the acute (model 1) and recovery (model 2) phases versus controls, all critical patients in the acute versus recovery phase (cross-sectional design, model 3), and paired critical patients in the acute versus recovery phase (longitudinal design, model 4). These four models were further repeated with non-critical patients (models 5 to 8) and reported in the Supplementary Material.

### 2.6 Statistical Analyses

Variables are reported as mean ± standard deviation for normally distributed continuous variables and median and interquartile range ([IQR], 25th to 75th percentile) for non-normally distributed continuous variables. Categorical variables are reported with frequencies and percentages. The Mann–Whitney U test was used to compare Raman peak intensities among the groups for features extracted by each RS-ML models. Additional Raman peak intensities were also compared among the groups to complement the molecular interpretation of each model and are reported in the Supplementary Material. Comparisons for age and sex among groups were performed using the Mann–Whitney U test and chi-square or Fisher’s exact test, respectively. Statistical significance was set at p<0.05.

## 3 Results

### 3.1 Clinical Characteristics and Demographics

Table 1 reports clinical characteristics and demographics of patients with COVID-19 during hospitalization (30 critical and 28 non-critical patients) and at the follow-up visit (10 critical and 11 non-critical patients) as well as in controls (n=30). Among the full cohort, 46 patients survived and were discharged home, whereas 12 patients died within DSO60.

**Table 1:** Clinical characteristics and demographics of COVID-19 patients during hospitalization (acute phase) and follow-up visit (recovery phase) and controls.

Variables	Hospitalization (acute phase)		Follow-up (recovery phase)		Controls (n=30)
	Critical (n=30)	Non-critical (n=28)	Critical (n=10)	Non-critical (n=11)	
Age, median (IQR), years	67 (57 to 75)**	52 (48 to 72)^‡^	59 (41 to 64)*	50 (43 to 54)^†^	41 (36 to 51)
Male sex, n (%)	19 (63)	15 (54)	7 (70)	6 (55)	16 (53)
DSO, median (IQR)	11 (10 to 13)	10 (9 to 13)	110 (74 to 123)	115 (62 to 126)	n/a
Symptoms, n (%)					
Cough^a^	18 (60)	18 (64)	0 (0)	0 (0)	0 (0)
Fever^b^	21 (70)	15 (54)	0 (0)	0 (0)	0 (0)
Dyspnea^b^	24 (80)	19 (68)	0 (0)	0 (0)	0 (0)
Anosmia^a^	1 (3)	2 (7)	0 (0)	0 (0)	0 (0)
Gut symptoms^c^	7 (23)	7 (25)	0 (0)	0 (0)	0 (0)
Respiratory support and hospital stay					
No oxygen, n (%)	0 (0)	15 (54)	0 (0)	0 (0)	n/a
Nasal canula, n (%)	0 (0)	13 (46)	0 (0)	0 (0)	n/a
Non-invasive, n (%)	12 (40)	0 (0)	0 (0)	0 (0)	n/a
Invasive, n (%)	18 (60)	0 (0)	0 (0)	0 (0)	n/a
ICU admission^d^, n (%)	21 (70)	4 (14)	0 (0)	0 (0)	n/a
Duration of intubation (days), median (IQR)	2 (0 to 20)	0 (0 to 0)	0 (0)	0 (0)	n/a
Duration of hospital stay (days)^b^, median (IQR)	17 (12 to 28)	10 (5 to 16)	0 (0)	0 (0)	n/a
Metabolic risk factors, n (%)					
Diabetes^d^	12 (40)	7 (25)	3 (30)	0 (0)	0 (0)
Obesity^e^	13 (43)	4 (14)	5 (6)	1 (9)	0 (0)
Overweight^e^	10 (33)	5 (18)	2 (20)	5 (45)	0 (0)
Hypertension^f^	15 (50)	12 (43)	4 (40)	2 (18)	0 (0)
Dyslipidemia^f^	15 (50)	5 (18)	4 (40)	0 (0)	0 (0)
Chronic diseases, n (%)					
Liver failure disease^d^	0 (0)	0 (0)	0 (0)	0 (0)	0 (0)
Renal failure disease^d^	0 (0)	1 (4)	0 (0)	0 (0)	0 (0)
Heart failure disease^d^	2 (7)	0 (0)	0 (0)	0 (0)	0 (0)
Respiratory disease^d^	3 (10)	4 (14)	0 (0)	1 (9)	0 (0)
Organ transplant^f^	2 (7)	0 (0)	1 (10)	0 (0)	0 (0)
Immunosuppression^d^	3 (10)	2 (7)	1 (10)	1 (9)	0 (0)
HIV	1 (3)	0 (0)	1 (10)	0 (0)	0 (0)
Resolved/active cancer^d^	2 (7)	2 (7)	1 (10)	0 (0)	0 (0)
Medication administration, n (%)					
Systematic corticosteroids (dexamethasone or hydrocortisone)	16 (53)	13 (43)	0 (0)	0 (0)	n/a
Tocilizumab	2 (6)	1 (5)	0 (0)	0 (0)	n/a
Outcome at DSO60, n (%)					
Survivors	19 (63)	27 (96)	10 (100)	11 (100)	n/a
Non-survivors	11 (37)	1 (4)	0 (0)	0 (0)	n/a

HIV, human immunodeficiency viruses; ICU, intensive care unit; IQR, interquartile range (25th to 75th percentile).

Age, sex, and DSO were available for all patients during the acute and recovery phases. During hospitalization, sample sizes were incomplete for the following variables:

a Cough and anosmia (n=48).

b Fever, dyspnea, and duration of hospital stay (n=50).

c Gut symptoms (n=43).

d Diabetes, liver failure, renal failure, heart failure, respiratory failure, immunosuppression, resolved/active cancer, and ICU admission (n=52).

e Obesity and overweight (n=47).

f Hypertension, dyslipidemia, and organ transplant (n=51).

Comparisons between critical (^*^p<0.05 and ^**^p<0.0001) and non-critical (^†^p<0.05 and ^‡^p<0.001) patients with controls.

### 3.2 RS-ML Model Performance

**Fig. 1:**
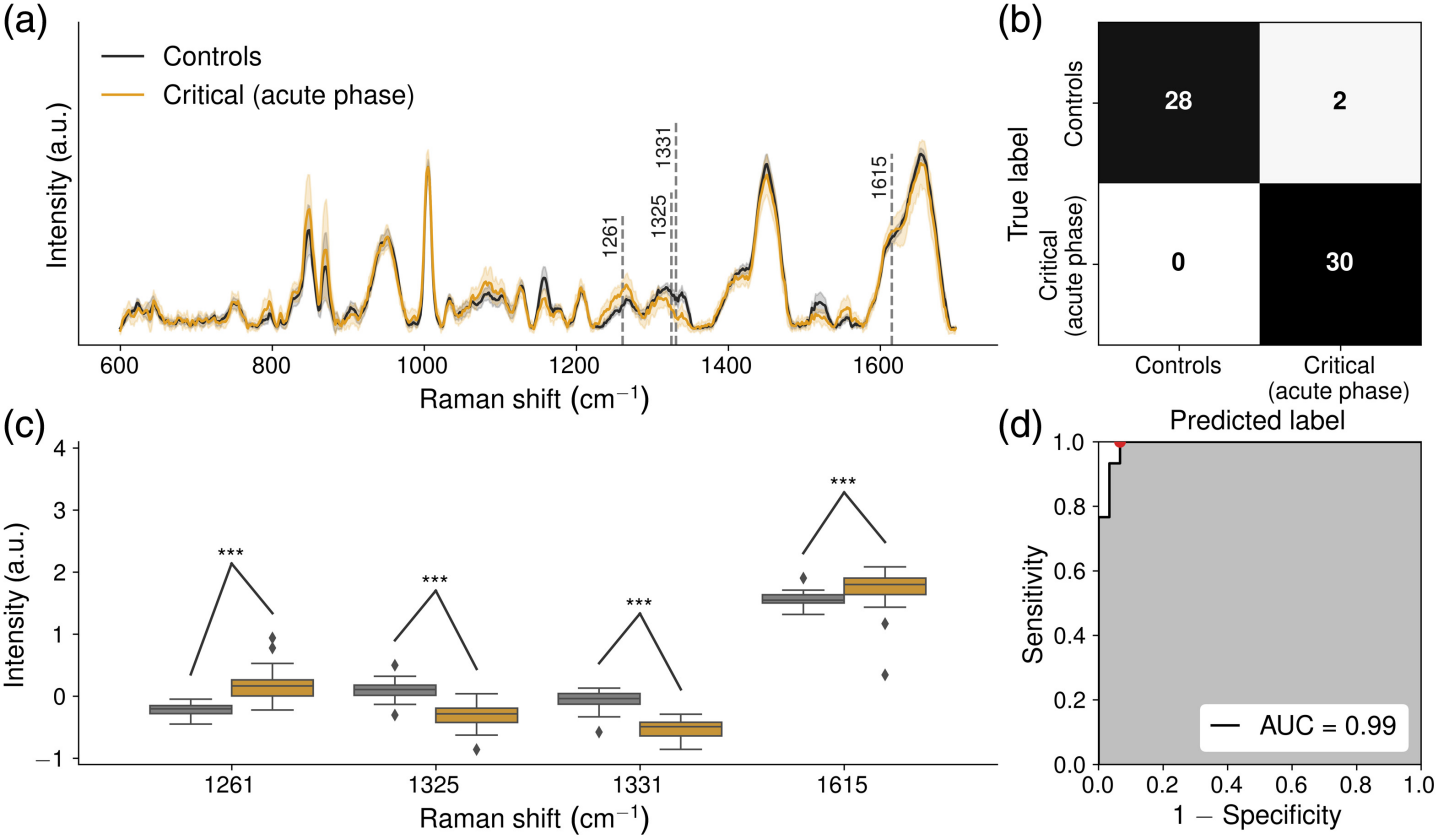
Model 1: critical patients in the acute phase (n=30, orange) versus controls (n=30, black). (a) Mean Raman spectra overlapped by variability and extracted features from the model (vertical dotted lines). (b) Confusion matrix. (c) Box-and-whisker plots showing the distribution of extracted Raman peaks from the model with outliers indicated as diamonds. (d) AUC. *p<0.05, **p<0.01, and ***p<0.001.

**Fig. 2:**
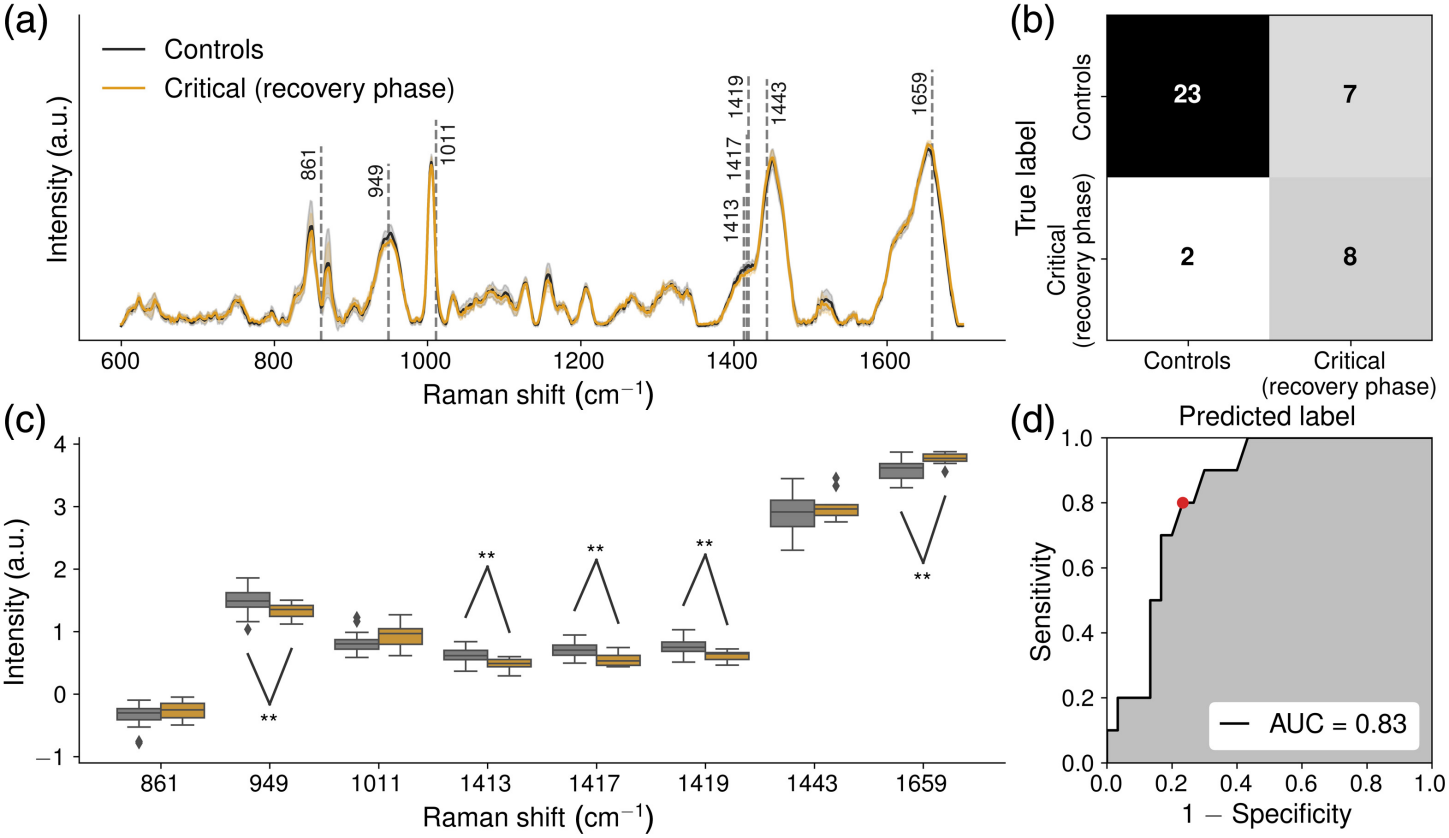
Model 2: critical patients in the recovery phase (n=10, orange) versus controls (n=30, black). (a) Mean Raman spectra overlapped by variability and extracted features from the model (vertical dotted lines). (b) Confusion matrix. (c) Box-and-whisker plots showing the distribution of extracted Raman peaks from the model with outliers indicated as diamonds. (d) AUC. *p<0.05, **p<0.01, and ***p<0.001.

**Fig. 3:**
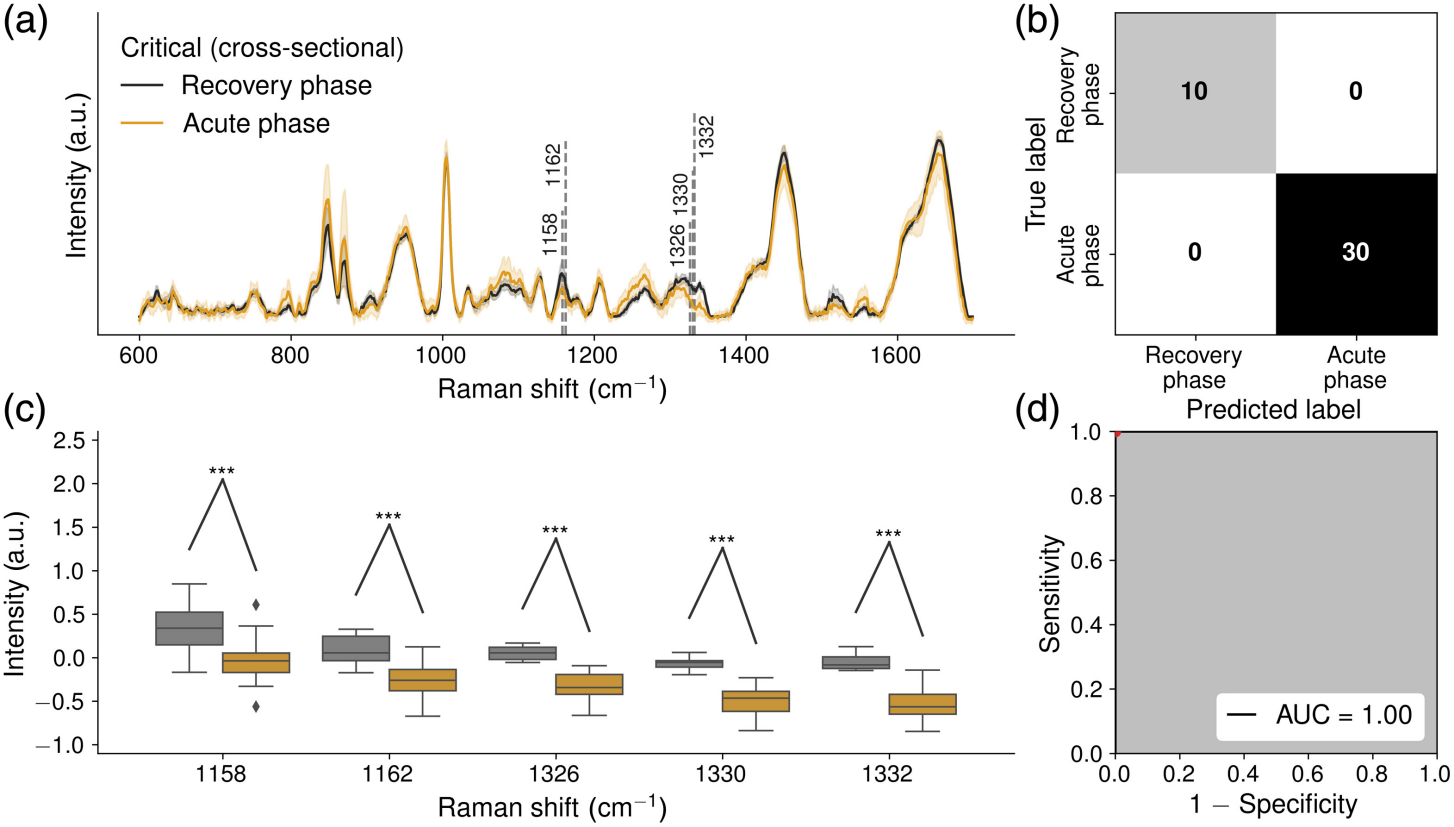
Model 3: critical patients in the acute (n=30, orange) versus recovery (n=10, black) phases (cross-sectional design). (a) Mean Raman spectra overlapped by variability and extracted features from the model (vertical dotted lines). (b) Confusion matrix. (c) Box-and-whisker plots showing the distribution of extracted Raman peaks from the model with outliers indicated as diamonds. (d) AUC. *p<0.05, **p<0.01, and ***p<0.001.

**Fig. 4:**
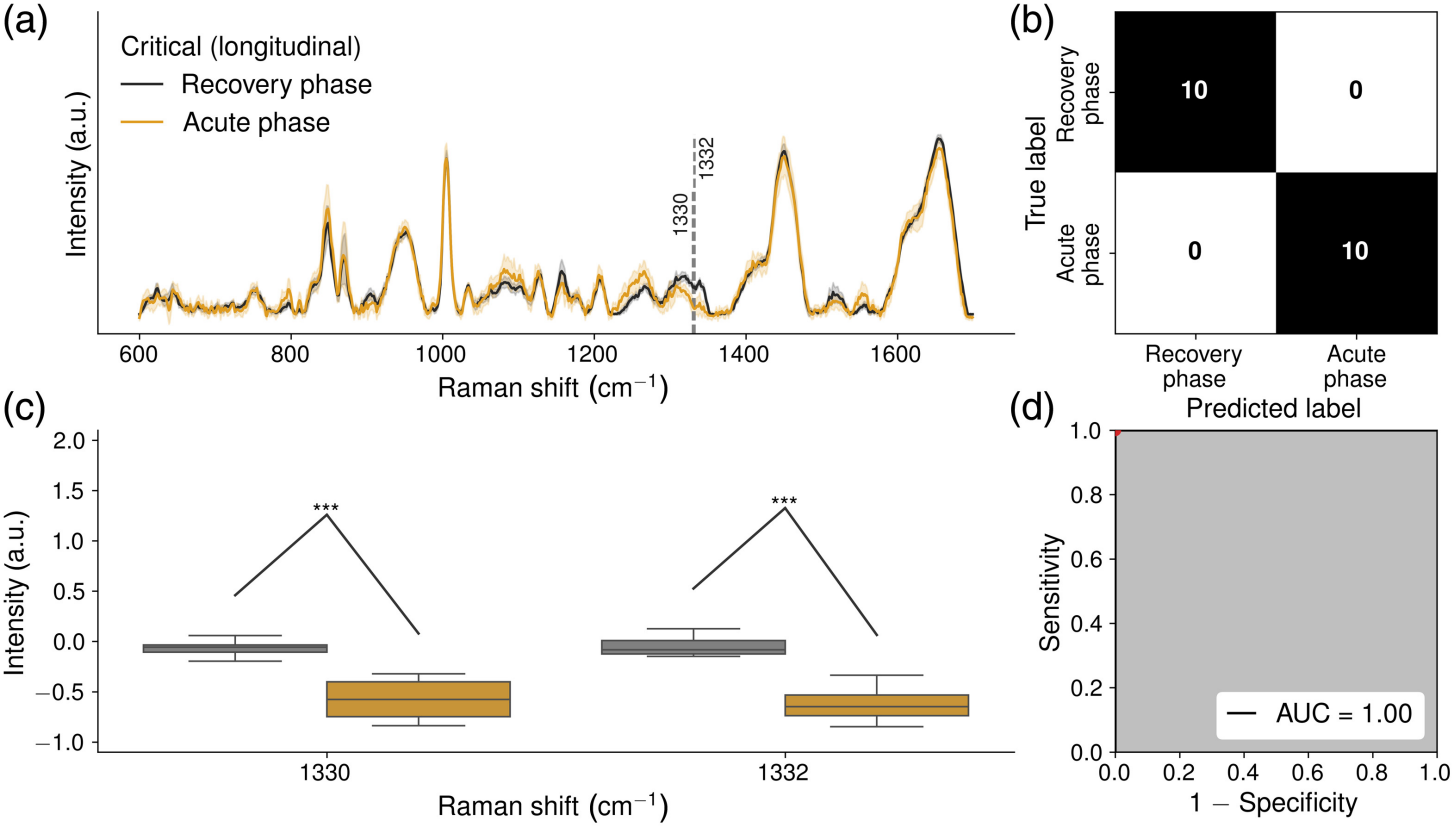
Model 4: paired critical patients in the acute (n=10, orange) versus recovery (n=10, black) phases (longitudinal design). (a) Mean Raman spectra overlapped by variability and extracted features from the model (vertical dotted lines). (b) Confusion matrix. (c) Box-and-whisker plots showing the distribution of extracted Raman peaks from the model with outliers indicated as diamonds. (d) AUC. *p<0.05, **p<0.01, and ***p<0.001.

Table 2 summarizes the performance of the eight ML models created to classify RS measurements of blood plasma samples. Figures 1–4 show RS-ML results in critical patients for models 1, 2, 3, and 4, respectively: mean Raman spectra with extracted key features highlighted by vertical dotted lines [panel (a)], confusion matrix [panel (b)], box-and-whisker plots showing the distribution of extracted features from the model [panel (c)], and corresponding AUC value with sensitivity and specificity [panel (d)]. Results from RS-ML models in non-critical patients (models 5 to 8) are reported in Figs. S1–S4 in the Supplementary Material.

**Table 2:** Machine learning models used to discriminate blood plasma from critical and non-critical COVID-19 patients during hospitalization (acute phase) and at follow-up (recovery phase), as well as from controls. Model performance was assessed by accuracy, specificity, sensitivity, and AUC value.

Model no.	Classification groups	Accuracy (%)	Specificity (%)	Sensitivity (%)	AUC
Critical patients					
1	Acute phase versus controls	97	93	100	0.99
2	Recovery phase versus controls	78	77	80	0.83
3	Acute versus recovery phase (cross-sectional)	100	100	100	1.00
4	Acute versus recovery phase (longitudinal)	100	100	100	1.00
Non-critical patients					
5	Acute phase versus controls	93	93	93	0.99
6	Recovery phase versus controls	83	87	73	0.87
7	Acute versus recovery phase (cross-sectional)	95	100	93	0.99
8	Acute versus recovery phase (longitudinal)	95	91	100	0.99

Models 1 and 2 built to discriminate critical patients in the acute and recovery phases versus controls achieved 97% and 78% accuracy, 93% and 77% specificity, 100% and 80% sensitivity, with an AUC of 0.99 and 0.83, respectively (Figs. 1 and 2, respectively). Models 3 and 4 built to classify critical patients in the acute versus recovery phase using the cross-sectional and longitudinal designs both achieved 100% accuracy, 100% specificity, 100% sensitivity, with an AUC of 1.00, respectively (Figs. 3 and 4, respectively).

Models 5 and 6 built to classify non-critical patients in the acute and recovery phases versus controls achieved 93% and 83% accuracy, 93% and 87% specificity, 93% and 73% sensitivity, with an AUC of 0.99 and 0.87, respectively (Figs. S1 and S2 in the Supplementary Material, respectively). Models 7 and 8 built to discriminate non-critical patients in the acute versus recovery phase using the cross-sectional and longitudinal designs achieved 95% accuracy, 100% and 91% specificity, 93% and 100% sensitivity, with an AUC of 0.99, respectively (Figs. S3 and S4 in the Supplementary Material, respectively).

### 3.3 Extracted Peaks by RS-ML Models

Table 3 provides the biomolecular assignments of Raman peaks extracted by the models, which were made through comparison with literature values.^51^^–^^62^ Among all models, 10 Raman features were extracted more than twice. Features located between 1300 and $1330\;{\mathrm{cm}}^{-1}$ were extracted by six models (models 1, 3, 4, 5, 7, and 8). These features are associated with the peak centered at $1318\;{\mathrm{cm}}^{-1}$ and assigned to protein (amide III, tryptophan, arginine, histidine, isoleucine, glutamate, glycine, tyrosine, valine, and C–H). Features located between 1331 and $1343\;{\mathrm{cm}}^{-1}$ were extracted by five models (models 1, 3, 4, 7, and 8). The associated Raman peak was centered at $1339\;{\mathrm{cm}}^{-1}$ and assigned to protein (amide III, arginine, aspartate, glycine, histidine, isoleucine, methionine, proline, valine, and C–H). In these cases, the corresponding Raman intensity signals were lower in patients (both critical and non-critical) in the acute phase compared with both the recovery phase and the controls.

Features located between 1149 and $1164\;{\mathrm{cm}}^{-1}$ were extracted by four models (models 3, 5, 7, and 8). These features are associated with the peak centered at $1157\;{\mathrm{cm}}^{-1}$ (vitamin A and fatty acids [C–C]). In all cases, the intensity of the Raman signal was lower in patients (both critical and non-critical) during the acute phase compared with both the recovery phase and control samples (except for model 8).

**Table 3:** Peak center, spectral range, and corresponding biomolecular assignments for Raman features extracted across all classification models. Amino acids listed alongside the proteins may either present freely in the blood plasma or be bound within protein structures. Assignments were made through comparison with literature values.^51^^–^^62^

Peak center (${\mathrm{cm}}^{-1}$)	Peak range (${\mathrm{cm}}^{-1}$)	Biomolecular assignments	Extracted by model no.
849	835 to 862	Protein (alanine, tyrosine, leucine, lysine, and proline) and lactate	2 and 8
903	896 to 910	Protein (C─H) and lipid (C─H)	5
952	941 to 963	Protein (tryptophan and valine) and citric acid	2 and 5
1005	998 to 1012	Protein (phenylalanine and tryptophan) and urea	2, 6, and 7
1157	1149 to 1164	Vitamin A and fatty acids (C─C)	3, 5, 7, and 8
1266	1257 to 1275	Protein (amide III, histidine, and valine), glucose, and lipid [═CH]	1 and 5
1318	1300 to 1330	Protein (amide III, tryptophan, arginine, histidine, isoleucine, glutamate, glycine, tyrosine, valine, and C─H)	1, 3, 4, 5, 7, and 8
1339	1331 to 1343	Protein (amide III, arginine, aspartate, glycine, histidine, isoleucine, methionine, proline, valine, and C─H)	1, 3, 4, 7, and 8
1419	1413 to 1422	Protein (amide I and C─H), lipid (${\mathrm{CH}}_{2}$), and DNA	2, 6, and 8
1451	1436 to 1466	Protein (amide I and C─H), lipid, and fatty acids (C─C)	2
1620	1600 to 1630	Protein (tyrosine, tryptophan, and phenylalanine)	1 and 5
1654	1631 to 1677	Lipid (C═ C) and protein (amide I)	2 and 6

Three models (models 2, 6, and 7) extracted the Raman peak centered at $1005\;{\mathrm{cm}}^{-1}$ (protein [phenylalanine and tryptophan] and urea) and at $1419\;{\mathrm{cm}}^{-1}$ (models 2, 6, and 8; assigned to protein [amide I and C─H], lipid [${\mathrm{CH}}_{2}$], and DNA). Raman intensity at 1005 and $1419\;{\mathrm{cm}}^{-1}$ was lower in the recovery phase (critical and non-critical patients) compared with controls. The following features were extracted twice: $849\;{\mathrm{cm}}^{-1}$ (models 2 and 8; protein [alanine, tyrosine, leucine, lysine, and proline], and lactate), $952\;{\mathrm{cm}}^{-1}$ (models 2 and 5; protein [tryptophan and valine] and citric acid), $1266\;{\mathrm{cm}}^{-1}$ (models 1 and 5; protein [amide III, histidine, and valine], glucose, and lipid [═CH]), $1620\;{\mathrm{cm}}^{-1}$ (models 1 and 5; protein [tyrosine, tryptophan, and phenylalanine]), and $1654{\;\mathrm{cm}}^{-1}$ (models 2 and 6; lipid [C═ C] and protein [amide I]). The peaks centered at $903\;{\mathrm{cm}}^{-1}$ (protein [C─H] and lipid [C─H]) and $1451\;{\mathrm{cm}}^{-1}$ (protein [amide I and C─H], lipid [${\mathrm{CH}}_{2}$], and DNA) were specific to models 5 and 2, respectively.

### 3.4 Group Comparison for Additional Raman Peaks Not Extracted by the RS-ML Models

Raman peaks that differed among groups but were not extracted by the RS-ML models are reported in Table S1 in the Supplementary Material. The peak centered at $796\;{\mathrm{cm}}^{-1}$ and assigned to glucose was higher in the acute phase compared with the recovery phase and controls in five group comparisons (corresponding to models 1, 3, 4, 5, and 7). Raman peak intensities associated with the peak centered at $903\;{\mathrm{cm}}^{-1}$ (protein [C─H] and lipid [C─H]) were lower in the acute phase compared with the recovery phase or controls, whereas the opposite trend was observed at 1085 (fatty acids [C─C]) and $1102\;{\mathrm{cm}}^{-1}$ (fatty acids [C─C], DNA, and RNA) in group comparisons corresponding to models 1, 3, and 4 (critical patients). For the peak centered at $624\;{\mathrm{cm}}^{-1}$ (protein [phenylalanine, glutamate, lysine, and tryptophan]), Raman intensity was lower in the acute phase compared with the recovery phase and controls, whereas it was the opposite at $1064\;{\mathrm{cm}}^{-1}$ (glucose, fatty acids [C─C], and lipids) in group comparisons from models 1 and 3 (critical patients). In group comparisons from models 1, 3, and 7, Raman intensities centered at $849\;{\mathrm{cm}}^{-1}$ (protein [alanine, tyrosine, leucine, lysine, and proline] and lactate) were higher in the acute phase compared with the recovery phase or controls. Raman intensity corresponding to the $1654\;{\mathrm{cm}}^{-1}$ peak (lipid [C═C] and protein [amide I]) was lower in the acute phase compared with the recovery phase in group comparisons from models 3, 4, and 7. The groups were different at other Raman peaks: 755 (model 7), 870 (models 7 and 8), 952 and 1127 (model 8), 1157 (models 1 and 4), 1266 (models 3 and 4), 1419 and 1451 (model 1), and $1620\;{\mathrm{cm}}^{-1}$ (models 5 and 6).

## 4 Discussion

In this study, we present a low-complexity, label-free blood plasma analysis approach using Raman spectroscopy combined with machine learning modeling. This technique allows differentiation between controls and COVID-19 patients who were in critical and non-critical conditions during the acute phase and at recovery. Extracted Raman peaks corresponded to proteins, glucose, fatty acids, lactic acid, vitamin A, and lipids. To the best of our knowledge, this study is the first to demonstrate the capability of RS-ML modeling to detect longitudinal biochemical perturbations induced by COVID-19 disease.

The eight classification models achieved AUC values between 0.83 and 1.00, with sensitivities, specificities, and accuracies ranging from 73% to 100%, 77% to 100%, and 78% to 100%, respectively. These results demonstrate that RS-ML can differentiate COVID-19 patients from controls during hospitalization and track temporal biomolecular changes. RS-ML could play a role in the monitoring of patient recovery even after hospital discharge.

The peaks centered at 1318 and $1339\;{\mathrm{cm}}^{-1}$ were critical to most of the RS-ML models differentiating between acute-phase COVID-19 blood plasma samples and other samples. In all cases, the intensity of this Raman signal was lower in acute phase samples compared with both the recovery phase and the controls. These peaks are primarily associated with protein, mostly aliphatic amino acids, C─H bonds, and the amide III region. A reduction in these peaks corresponds to current COVID-19 infection but does not correspond to COVID-19 severity.^33^ Furthermore, prior studies using mass spectrometry demonstrated that COVID-19 patients exhibit alterations in circulating amino acid levels, potentially reflecting changes in cellular metabolism and protein turnover in response to metabolic demands.^60^^–^^62^ Such changes in circulating amino acids (and therefore availability of amino acids for protein synthesis) could be responsible for these Raman spectral differences.

The next most common peak used by the ML models was the one centered at $1157\;{\mathrm{cm}}^{-1}$ (vitamin A and fatty acids [C─C]). In all cases, the intensity of the Raman signal was lower in samples taken during the acute phase compared with other samples, indicating a lower relative circulating concentration of either vitamin A and/or fatty acids. In another Raman-based study, a reduction in this peak was associated with lung cancer presence.^63^ Also, non-Raman-based studies have shown that there is a reduction in circulating vitamin A concentrations (specifically retinol) associated with COVID-19 infection and other infectious diseases.^64^^–^^66^

Models 1 and 5 demonstrated the potential of RS-ML to differentiate between controls and COVID-19 acute phase samples in critical and non-critical patients, respectively. This was achieved for both models with an AUC of 0.99, demonstrating that there is a specific Raman signature in blood plasma associated with acute COVID-19 infection. In contrast to the acute phase models, models 2 and 6 differentiated controls from recovered critical patients at the follow-up visit. The lower AUC values (0.83 and 0.87) compared with acute phase detection models likely reflect the absence of the pronounced immunological and metabolic alterations typically associated with active disease. At follow-up, patients were unlikely to have ongoing COVID-19 infection. Nevertheless, some differences in the molecular profile of blood plasma were still observed compared with controls. These persistent alterations may be related, at least in part, to post-intensive care syndrome.^67^^,^^68^ As the peak at $1654\;{\mathrm{cm}}^{-1}$ is sharper in lipids than in proteins, it is possible that circulating concentration of some lipids is higher in critical patients at recovery than controls, which is consistent with plasma metabolomics of long-COVID patients. Our approach could pave the way for long-COVID detection/monitoring.^69^

Extending the analysis to temporal comparisons (cross-sectional design), models 3 and 7 differentiated acute infection and recovery status in critical and non-critical patients, respectively. The higher AUC values observed in these models (1.00 and 0.99), compared with those of model 2 (0.83) and model 6 (0.87), suggest that the molecular profile of blood plasma in recovered critical and non-critical patients is more similar to that of controls than during the acute phase of infection. To further account for inter-patient variability, models 4 and 8 were developed using paired longitudinal samples, enabling direct within-patient comparison of acute infection and recovery status. Model 4 achieved an AUC of 1.00 and was based on only two features within two consecutive discriminative peaks (1318 and $1339\;{\mathrm{cm}}^{-1}$). Model 8 achieved an AUC of 0.99 using six features. The slightly lower AUC and greater number of features compared with model 4 likely reflect the closer similarity between the acute and recovered states in non-critical patients, in whom biomolecular changes were less pronounced than in critical patients during the acute phase. Overall, models 3, 4, 7, and 8 highlight the potential of RS-ML as a longitudinal monitoring tool for assessing patient status and disease progression, even after hospital discharge.

All features extracted by the critical acute versus control RS-ML model (model 1) were found within the same peaks as the features extracted by the corresponding non-critical model (model 5). Consistent with these observations, model 5 differentiated non-critical patients in the acute phase from controls with an accuracy comparable to that of model 1. However, it relied on a greater number of features (8 versus 4), which may reflect less pronounced biomolecular differences between non-critical patients and controls. Furthermore, all features extracted by the non-critical recovery phase versus control RS-ML model (model 6) were found within the same peaks as the features extracted by the corresponding critical model (model 2). All features extracted by the critical acute phase versus recovery phase RS-ML models (models 3 and 4) were found within the same peaks as the features extracted by the non-critical acute phase versus recovery phase RS-ML models (models 7 and 8). These results suggest that there are common biomolecular signatures of COVID-19 presence regardless of the severity of infection.

Our approach has potential clinical impacts as a rapid and accurate method for characterizing the progression of COVID-19 disease. Sample analysis was achieved using a small volume of liquid blood plasma, eliminating the need for a sample drying step, and no external reagents, reducing sample processing steps and costs. Time from sample thawing to spectra measurement was 60 min. The RS system used in this study is small enough to fit within an enclosure placed on a cart, allowing it to be wheeled into hospitals, doctors’ offices, or pharmacies. The total cost of the system is lower than that of commercial Raman microscopes. These characteristics are relevant when considering a clinical implementation.

A primary limitation of this study lies in the modest dataset sizes, in particular, at the follow-up visit, which may lead to potential overfitting. However, specific strategies were used to minimize this potential overfitting: (1) the dimensionality of the models was proactively reduced by removing irrelevant or redundant features through the application of the L1-regularization SVM to focus only on the most important features (the subset of 26 peaks), (2) a gradient boosting approach was selected to further minimize this overfitting through ensemble averaging and regularization,^48^ and (3) a fivefold cross-validation was performed to ensure that model estimation and evaluation are assessed on independent subsets to preserve generalization. RS measurements were repeated at a single spot, which may not reflect the full biomolecular composition of the sample. However, plasma was vortexed prior to data acquisition, which allowed to increase molecular motion and capture molecules in different conformations and spatial distributions. Our RS-ML approach, based on spectral standardization and high-SNR data acquisition, enabled the identification of key discriminating features among the groups despite the complexity of direct spectral interpretation. The Raman-derived features represent integrated biochemical signatures rather than quantitative measurements of individual metabolites, consistent with the composite nature of Raman signals in complex biofluids.

## 5 Conclusion

The aim of this study was to combine label-free RS with ML modeling to monitor the disease course of COVID-19 and identify biochemical dysregulation in patients. Our approach allowed us to discriminate the acute (hospitalization) and recovery (follow-up) phases of critical and non-critical patients and differentiate them from controls with AUC values ranging between 0.83 and 1.00 with corresponding sensitivities, specificities, and accuracies of 73% to 100%, 77% to 100%, and 78% to 100%, respectively. Follow-up profiles suggested progressive recovery, with the main molecular differences observed in proteins, glucose, fatty acids, lactic acid, vitamin A, and lipids. These changes indicate that the Raman spectral profiles at follow-up had shifted toward those of controls but had not fully recovered even months after symptom onset, suggesting that additional factors may influence recovery. Overall, the results presented in this study provide evidence that RS-ML modeling can be utilized for rapid biofluid classification and biochemical analysis in infectious disease screening. Future studies will be carefully designed to ensure that these models are trained on data that fully represent the heterogeneity of both disease pathology and the general population.

## Supplementary Material

10.1117/1.JBO.31.7.077002.s01

## Data Availability

The material that supports the findings of this study is available from the corresponding author upon reasonable request. Figures S1–S4 and Table S1 in the Supplementary Material provide additional supporting information related to this study.
