# Supplementary material for: Combining label-free Raman spectroscopy with machine learning to monitor COVID-19 disease from acute infection to recovery
Source: J Biomed Opt. 2026 Jul 20;31(7):077002. doi: 10.1117/1.JBO.31.7.077002 (PMC13384748; doi:10.1117/1.JBO.31.7.077002)
Supplement: Supplementary file 1 [file JBO_031_077002_SD001.docx]

***Combining Label-Free Raman Spectroscopy with Machine Learning to Monitor COVID-19 Disease from Acute Infection to Recovery***

**Maryam Heidarifard^a,b,c^, Frédéric Leblond^b,d^, Frédérick Dallaire^b,d^, Elsa Brunet-Ratnasingham^b,e^, Nassim Ksantini^b,d^, Myriam Mahfoud^b,d^, Guillaume Sheehy^b,d^, Hugo Soudeyns^a,f,g^, Philippe Jouvet^a,h^, Sze Man Tse^a,h^, Caroline Quach^a,i^, Daniel E. Kaufmann^i,j,k,#^, Katherine Ember^b,d,#^ and Mathieu Dehaes^a,c,l,#,*^**

^#^Equal contribution as co-senior authors

^a^Centre de recherche Azrieli du CHU Sainte-Justine, Montreal, Quebec, Canada

^b^Research Centre, CHU Montreal, Montreal, Quebec, Canada

^c^Institute of Biomedical Engineering, University of Montreal, Montreal, Quebec, Canada

^d^Department of Engineering Physics, Polytechnique Montreal, Montreal, Quebec, Canada

^e^Department of Microbiology, Infectiology and Immunology, University of Montreal, Montreal, Quebec, Canada

^f^Department of Pathology and Laboratory Medicine, Pathology Advanced Translational

^g^Research Unit, Emory University School of Medicine, Atlanta, Georgia, United States

^h^Department of Pediatrics, University of Montreal, Montreal, Quebec, Canada

^i^Department of Microbiology, Infectious Diseases and Immunology, University of Montreal, Montreal, Quebec, Canada

^j^Division of Infectious Diseases, Lausanne University Hospital and University of Lausanne, Vaud, Switzerland

^k^Department of Medicine, University of Montreal, Montreal, Quebec, Canada

^l^Department of Radiology, Radio-oncology and Nuclear Medicine, University of Montreal, Montreal, Quebec, Canada

**^*^**Corresponding Author, E-mail: [mathieu.dehaes@umontreal.ca](mailto:mathieu.dehaes@umontreal.ca)

**Supplementary Material**

| **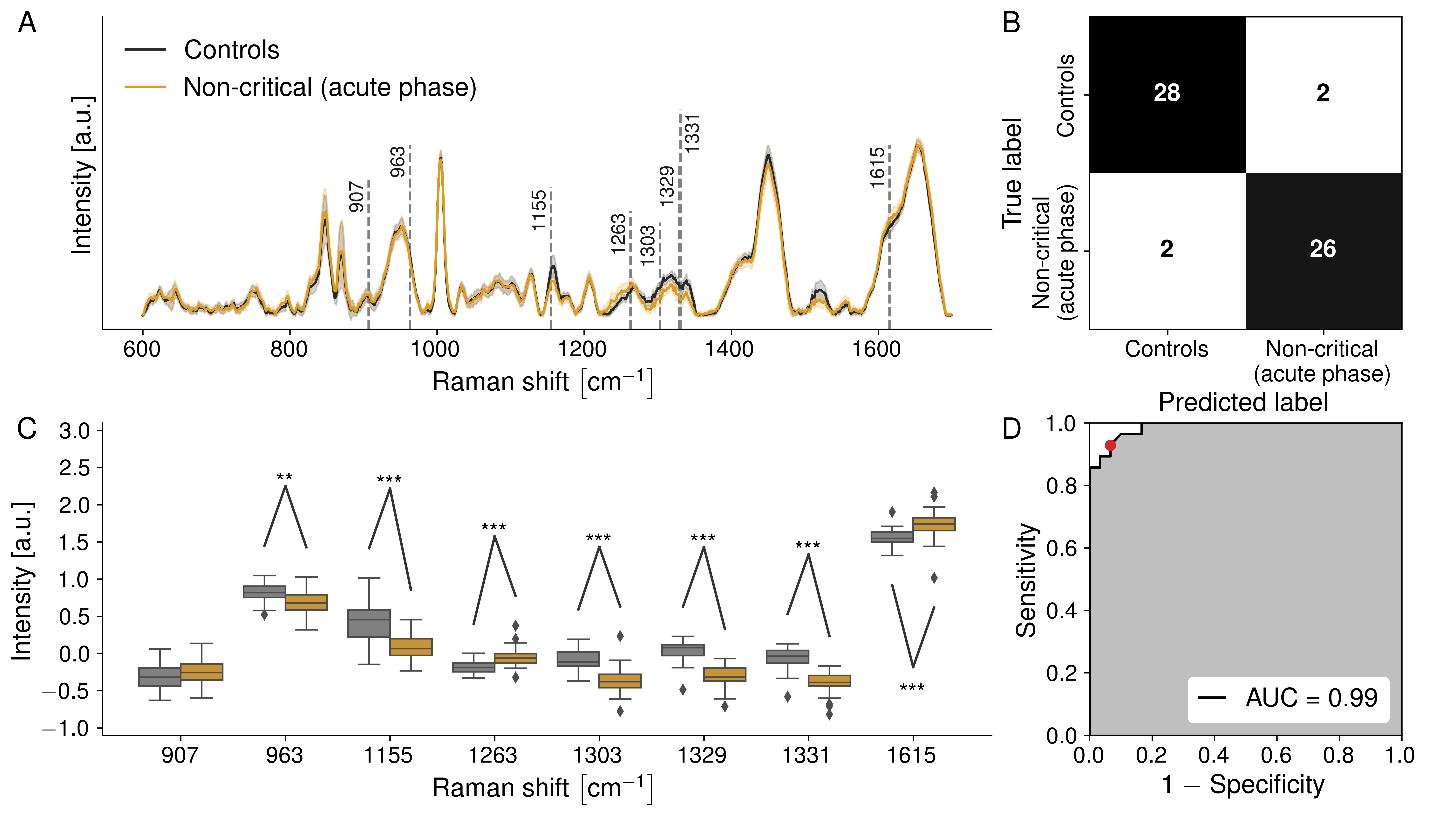** |
| --- |

**Supplemntary Figure S1.** Model 5: Non-critical patients in the acute phase (n=28, orange) vs. controls (n=30, black). (A) Mean Raman spectra overlapped by variability and extracted features from the model (vertical dotted lines), (B) confusion matrix, (C) area under the receiver operating characteristic curve (AUC) and (D) box-and-whisker plots showing the distribution of extracted Raman peaks from the model and additional features, with outliers indicated as diamonds. Significance levels are indicated as ^*^*p* < 0.05, ^**^*p* < 0.01, ^***^*p* < 0.001.

| **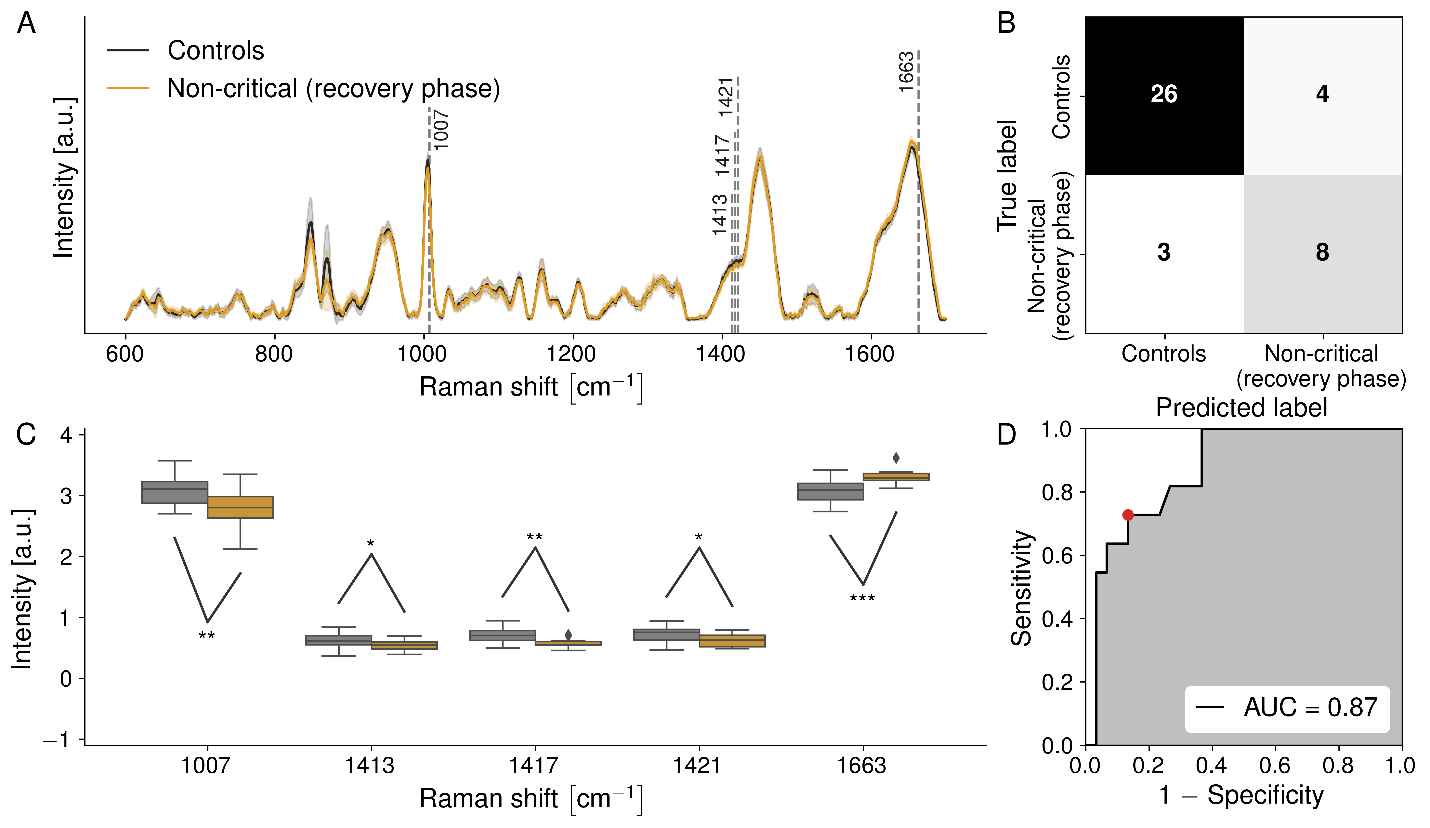** |
| --- |

**Supplementary Figure S2.** Model 6: Non-critical patients in the recovery phase (n=11, orange) vs. controls (n=30, black). (A) Mean Raman spectra overlapped by variability and extracted features from the model (vertical dotted lines), (B) confusion matrix, (C) area under the receiver operating characteristic curve (AUC) and (D) box-and-whisker plots showing the distribution of extracted Raman peaks from the model and additional features, with outliers indicated as diamonds. Significance levels are indicated as ^*^*p* < 0.05, ^**^*p* < 0.01.

**
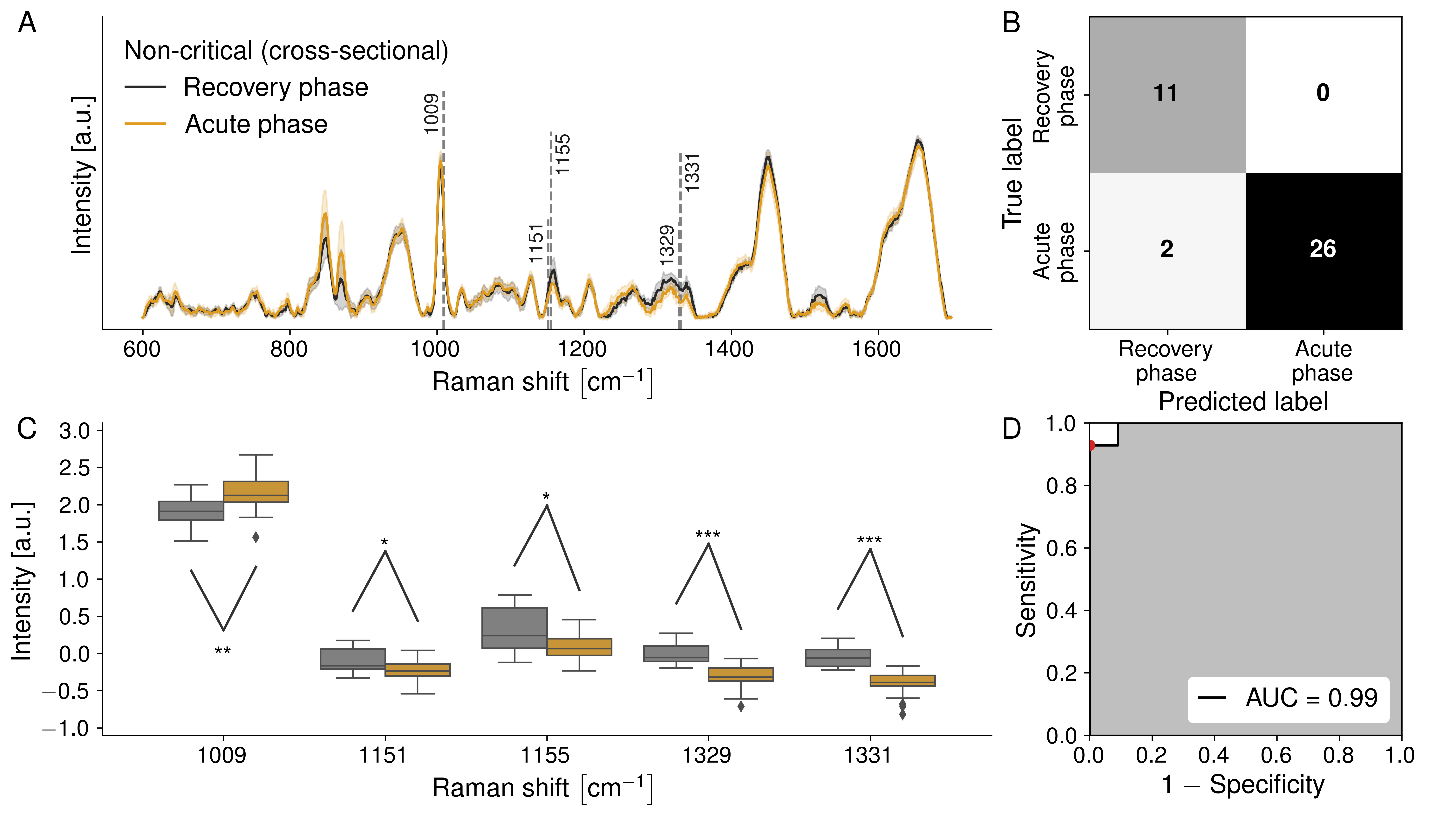
**

**Supplementary Figure S3.** Model 7: Non-critical patients in the acute (n=28, orange) vs. recovery (n=11, black) phase (cross-sectional design). (A) Mean Raman spectra overlapped by variability and extracted features from the model (vertical dotted lines), (B) confusion matrix, (C) area under the receiver operating characteristic curve (AUC) and (D) box-and-whisker plots showing the distribution of extracted Raman peaks from the model and additional features, with outliers indicated as diamonds. Significance levels are indicated as ^*^*p* < 0.05, ^**^*p* < 0.01, ^***^*p* < 0.001.

*
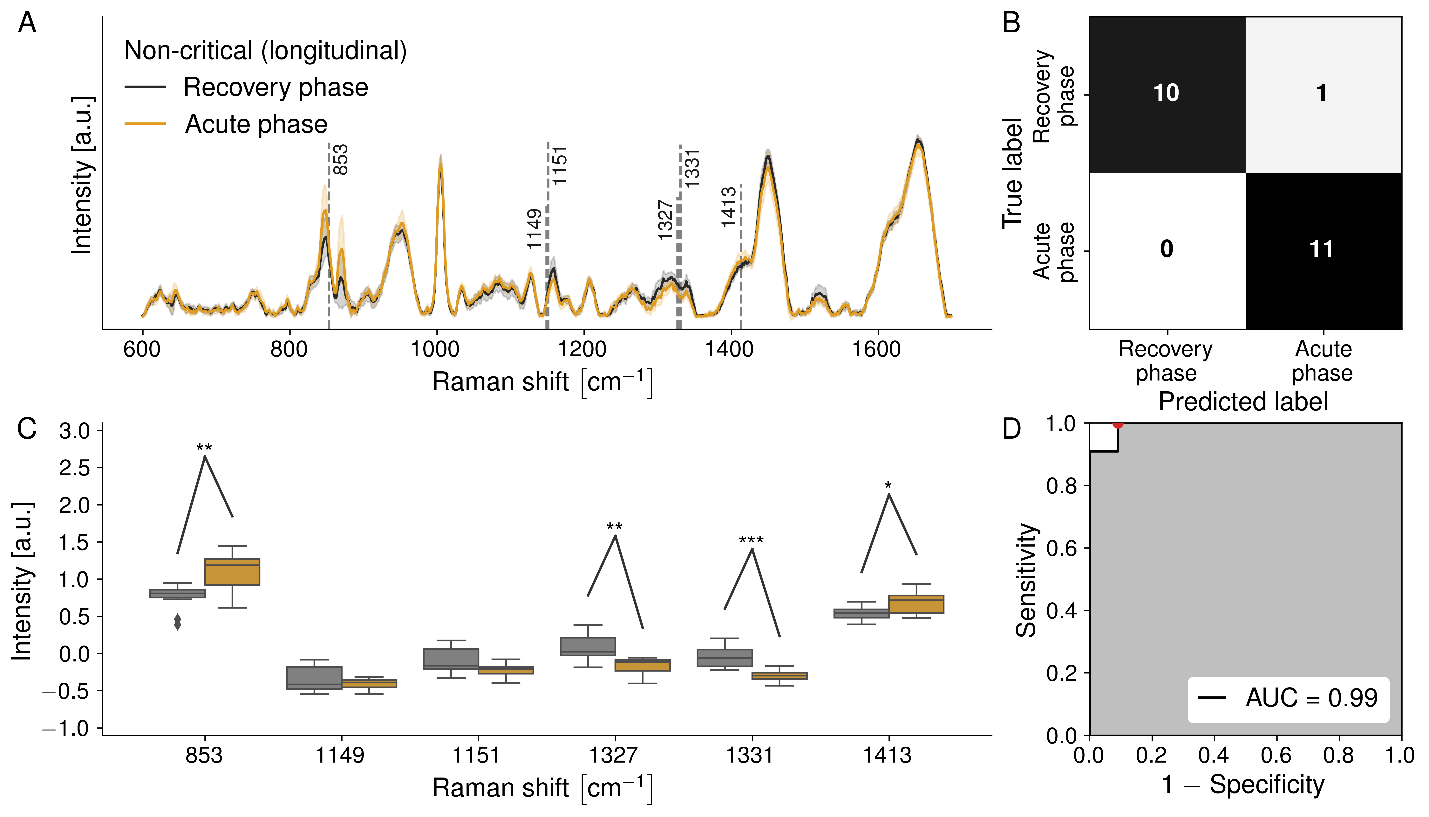
*

**Supplementary Figure S4.** Model 8: Paired non-critical patients in the acute (n=11, orange) vs. recovery (n=11, black) phase (longitudinal design). (A) Mean Raman spectra overlapped by variability and extracted features from the model (vertical dotted lines), (B) confusion matrix, (C) area under the receiver operating characteristic curve (AUC) and (D) box-and-whisker plots showing the distribution of extracted Raman peaks from the model and additional features, with outliers indicated as diamonds. Significance levels are indicated as ^*^*p* < 0.05, ^**^*p* < 0.01.

**Supplementary Table S1.** Peak center, spectral range, mean and standard deviation per groups, and corresponding biomolecular assignments for Raman features that were not extracted by the RS-ML models. Amino acids listed alongside the proteins may either present freely in the blood plasma or be bound within protein structures. Assignments were made from literature values. [^1-12^](#_ENREF_1)

| **Critical patients** | | | | | |
| --- | --- | --- | --- | --- | --- |
| **Model 1** | **Peak center (cm^-1^)** | **Peak range**  **(cm^-1^)** | **Acute phase** | **Controls** | **Biomolecular assignments** |
|  | 624 | 618-630 | -0.286 ± 0.221 | -0.166 ± 0.116** | Protein (phenylalanine, glutamate, lysine, tryptophan) |
|  | 796 | 788-804 | -0.113 ± 0.314*** | -0.428 ± 0.130 | Glucose |
|  | 849 | 835-863 | 2.289 ± 0.866* | 1.753 ± 0.627 | Protein (alanine, tyrosine, leucine, lysine, and proline), and lactate |
|  | 903 | 896-910 | -0.458 ± 0.294 | -0.265 ± 0.169** | Protein [C-H], lipid [C-H] |
|  | 1064 | 1059-1069 | 0.053 ± 0.207*** | -0.116 ± 0.156 | Glucose, fatty acids (C-C), lipids |
|  | 1085 | 1073-1097 | 0.377 ± 0.344*** | 0.121 ± 0.201 | Fatty acids (C-C) |
|  | 1102 | 1095-1109 | 0.204 ± 0.267** | 0.045 ± 0.196 | Fatty acids (C-C), DNA, RNA |
|  | 1157 | 1150-1164 | -0.045 ± 0.254 | 0.507 ± 0.298*** | Vitamin A, fatty acids [C-C] |
|  | 1419 | 1413-1422 | 0.562 ± 0.219*** | 0.751 ± 0.125 | Protein (amide I, C-H), lipid [CH₂], DNA |
|  | 1451 | 1436-1466 | 3.197 ± 0.544 | 3.483 ± 0.269* | Protein (amide I, C-H), lipids, fatty acids (C-C) |
| **Model 3** | **Peak center (cm^-1^)** | **Peak range**  **(cm^-1^)** | **Cross-sectional design** | | **Biomolecular assignments** |
|  |  |  | **Acute**  **phase** | **Recovery phase** |  |
|  | 624 | 618-630 | -0.286 ± 0.221 | -0.090 ± 0.114** | Protein (phenylalanine, glutamate, lysine, tryptophan) |
|  | 796 | 788-804 | -0.113 ± 0.314*** | -0.481 ± 0.086 | Glucose |
|  | 849 | 835-863 | 2.289 ± 0.866 | 1.635 ± 0.442* | Protein (alanine, tyrosine, leucine, lysine, and proline), and lactate |
|  | 903 | 896-910 | -0.458 ± 0.294 | -0.262 ± 0.168* | Protein [C-H], lipid [C-H] |
|  | 1064 | 1059-1069 | 0.053 ± 0.207* | -0.126 ± 0.162 | Glucose, fatty acids (C-C), lipids |
|  | 1085 | 1073-1097 | 0.377 ± 0.344** | 0.049 ± 0.114 | Fatty acids (C-C) |
|  | 1102 | 1095-1109 | 0.204 ± 0.267** | -0.042 ± 0.118 | Fatty acids (C-C), DNA, RNA |
|  | 1266 | 1257-1275 | 0.322 ± 0.279 | -0.034 ± 0.073*** | Protein (amide III, histidine, valine), glucose and lipid [=CH] |
|  | 1654 | 1631-1677 | 3.497 ± 0.584 | 3.867 ± 0.109* | Lipid [C=C], protein (amide I) |
| **Model 4** | **Peak center (cm^-1^)** | **Peak range**  **(cm^-1^)** | **Longitudinal design** | | **Biomolecular assignments** |
|  |  |  | **Acute phase** | **Recovery phase** |  |
|  | 796 | 788-804 | -0.124 ± 0.280** | -0.481 ± 0.086 | Glucose |
|  | 903 | 896-910 | -0.521 ± 0.263 | -0.262 ± 0.168* | Protein [C-H], lipid [C-H] |
|  | 1085 | 1073-1097 | 0.380 ± 0.361* | 0.049 ± 0.114 | Fatty acids (C-C) |
|  | 1102 | 1095-1109 | 0.260 ± 0.274** | -0.042 ± 0.118 | Fatty acids (C-C), DNA, RNA |
|  | 1157 | 1150-1164 | 0.045 ± 0.240 | 0.364 ± 0.305* | Vitamin A, fatty acids [C-C] |
|  | 1266 | 1257-1275 | 0.359 ± 0.280** | -0.034 ± 0.073 | Protein (amide III, histidine, valine), glucose and lipid [=CH] |
|  | 1654 | 1631-1677 | 3.588 ± 0.238 | 3.867 ± 0.109** | Lipid [C=C], protein (amide I) |
| **Non-critical patients** | | | | | |
| **Model 5** | **Peak center (cm^-1^)** | **Peak range**  **(cm^-1^)** | **Acute**  **phase** | **Controls** | **Biomolecular assignments** |
|  | 796 | 788-804 | -0.301 ± 0.159** | -0.428 ± 0.130 | Glucose |
|  | 1451 | 1436-1466 | 3.232 ± 0.368 | 3.483 ± 0.269** | Protein (amide I, C-H), lipids, fatty acids (C-C) |
|  | 1620 | 1600-1630 | 1.843 ± 0.209*** | 1.670 ± 0.156 | Protein (tyrosine, tryptophan, phenylalanine) |
| **Model 6** | **Peak center (cm^-1^)** | **Peak range**  **(cm^-1^)** | **Recovery phase** | **Controls** | **Biomolecular assignments** |
|  | 1620 | 1600-1630 | 1.759 ± 0.122* | 1.670 ± 0.156 | Protein (tyrosine, tryptophan, phenylalanine) |
| **Model 7** | **Peak center (cm^-1^)** | **Peak range**  **(cm^-1^)** | **Cross-sectional design** | | **Biomolecular assignments** |
|  |  |  | **Acute**  **phase** | **Recovery phase** |  |
|  | 755 | 741-769 | -0.084 ± 0.137* | -0.177 ± 0.071 | Protein (tryptophan, phenylalanine, aspartate, isoleucine) |
|  | 796 | 788-804 | -0.301 ± 0.159* | -0.432 ± 0.106 | Glucose |
|  | 849 | 835-863 | 1.970 ± 0.587** | 1.322 ± 0.518 | Protein (alanine, tyrosine, leucine, lysine, and proline), and lactate |
|  | 870 | 863-877 | 0.911 ± 0.827* | 0.222 ± 0.777 | Fatty acids, lipids (C-O-O), protein (tyrosine, arginine, aspartate, glutamate, isoleucine, methionine, threonine, tryptophan) |
|  | 1654 | 1631-1677 | 3.761 ± 0.217 | 3.925 ± 0.127* | Lipid [C=C], protein (amide I) |
| **Model 8** | **Peak center (cm^-1^)** | **Peak range**  **(cm^-1^)** | **Longitudinal design** | | **Biomolecular assignments** |
|  |  |  | **Acute**  **phase** | **Recovery phase** |  |
|  | 870 | 863-877 | 0.960 ± 0.853* | 0.222 ± 0.777 | Fatty acids, lipids (C-O-O), protein (tyrosine, arginine, aspartate, glutamate, isoleucine, methionine, threonine, tryptophan) |
|  | 952 | 941-963 | 1.693 ± 0.346* | 1.491 ± 0.228 | Protein (tryptophan, valine), citric acid |
|  | 1127 | 1119-1135 | 0.358 ± 0.172* | 0.234 ± 0.114 | Glucose, lipids (triacylglycerides, C-C), protein (C-N) |

**Notes:** Model 2 did not show additional peak differences between groups. Significance levels are indicated as ^*^*p* < 0.05, ^**^*p* < 0.01, ^***^*p* < 0.001.

**Refrences:**

1. C. G. Atkins, K. Buckley, M. W. Blades and R. F. B. Turner, "Raman Spectroscopy of Blood and Blood Components," *Appl Spectrosc* **71**(5), 767-793 (2017). <https://doi.org/10.1177/0003702816686593>.

2. J. Y. Qu, B. C. Wilson and D. Suria, "Concentration measurements of multiple analytes in human sera by near-infrared laser Raman spectroscopy," *Appl Opt* **38**(25), 5491-5498 (1999). <https://doi.org/10.1364/ao.38.005491>.

3. A. J. Berger et al., "Multicomponent blood analysis by near-infrared Raman spectroscopy," *Appl Opt* **38**(13), 2916-2926 (1999). <https://doi.org/10.1364/ao.38.002916>.

4. A. Rygula et al., "Raman spectroscopy of proteins: a review," *Journal of Raman Spectroscopy* **44**(8), 1061-1076 (2013).

5. K. Czamara et al., "Raman spectroscopy of lipids: a review," *Journal of Raman spectroscopy* **46**(1), 4-20 (2015).

6. G. Zhu, X. Zhu, Q. Fan and X. Wan, "Raman spectra of amino acids and their aqueous solutions," *Spectrochim Acta A Mol Biomol Spectrosc* **78**(3), 1187-1195 (2011). <https://doi.org/10.1016/j.saa.2010.12.079>.

7. A. Falamas, S. Kalra, V. Chis and I. Notingher, "Monitoring the RNA distribution in human embryonic stem cells using Raman micro-spectroscopy and fluorescence imaging," *AIP Conference Proceedings* 43-47 (2013).

8. J. Shao et al., "In vivo blood glucose quantification using Raman spectroscopy," *PLoS One* **7**(10), e48127 (2012). <https://doi.org/10.1371/journal.pone.0048127>.

9. Z. Huang et al., "Quantitative determination of citric acid in seminal plasma by using Raman spectroscopy," *Appl Spectrosc* **67**(7), 757-760 (2013). <https://doi.org/10.1366/12-06902>.

10. Z. Movasaghi, S. Rehman and I. U. Rehman, "Raman spectroscopy of biological tissues," *Applied Spectroscopy Reviews* **42**(5), 493-541 (2007).

11. M. Baranska et al., "Recent advances in Raman analysis of plants: alkaloids, carotenoids, and polyacetylenes," *Current Analytical Chemistry* **9**(1), 108-127 (2013).

12. C. G. Angiboust, L. Van-Gulick, P. Jeannesson and O. Piot, "Probing In Vitro Ribose Induced DNA-Glycation Using Raman Microspectroscopy."
